# Loss of UBE3A from TH-expressing neurons suppresses GABA co-release and enhances VTA-NAc optical self-stimulation

**DOI:** 10.1038/ncomms10702

**Published:** 2016-02-12

**Authors:** Janet Berrios, Alice M. Stamatakis, Pranish A. Kantak, Zoe A. McElligott, Matthew C. Judson, Megumi Aita, Marie Rougie, Garret D. Stuber, Benjamin D. Philpot

**Affiliations:** 1 Curriculum in Neurobiology, University of North Carolina, Chapel Hill, 27599 North Carolina, USA; 2 Department of Cell Biology and Physiology, University of North Carolina, Chapel Hill, 27599 North Carolina, USA; 3 Neuroscience Center, University of North Carolina, Chapel Hill, 27599 North Carolina, USA; 4 Department of Psychiatry, University of North Carolina, Chapel Hill, 27599 North Carolina, USA; 5 Inscopix Inc, Palo Alto, 94303 California, USA; 6 Bowles Center for Alcohol Studies, University of North Carolina, Chapel Hill, 27599 North Carolina, USA; 7 Carolina Institute for Developmental Disabilities, University of North Carolina, Chapel Hill, 27599 North Carolina, USA

## Abstract

Motivated reward-seeking behaviours are governed by dopaminergic ventral tegmental area projections to the nucleus accumbens. In addition to dopamine, these mesoaccumbal terminals co-release other neurotransmitters including glutamate and GABA, whose roles in regulating motivated behaviours are currently being investigated. Here we demonstrate that loss of the E3-ubiquitin ligase, UBE3A, from tyrosine hydroxylase-expressing neurons impairs mesoaccumbal, non-canonical GABA co-release and enhances reward-seeking behaviour measured by optical self-stimulation.

## Abstract (web summary)

Mesoaccumbal terminals within the VTA are known to co-release both GABA and dopamine, although the functional role of the former has yet to be determined. Here, the authors find that non-canonical GABA release is regulated by the E3-ubiquitin ligase, UBE3A, and enhances optogenetic self-stimulation.

## Graphical abstract

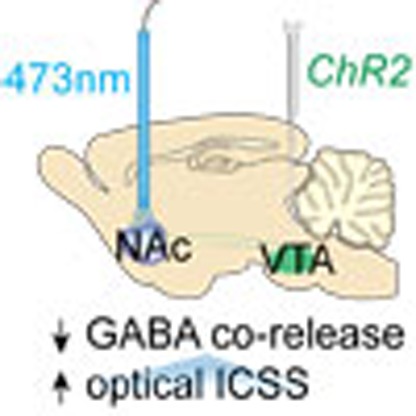

Dopamine projections from the ventral tegmental area (VTA) target the nucleus accumbens (NAc) and release dopamine in response to reward-predictive cues, which in turn initiates reward-seeking and promotes reward learning^1^,^2^,^3^. While phasic dopamine dynamics have been well-characterized within the NAc^1^, dopaminergic terminals are now known to be capable of co-releasing several neurotransmitters including gamma-aminobutyric acid (GABA) and glutamate^4^,^5^,^6^,^7^. However, the role of mesoaccumbal non-canonical GABA and glutamate co-release remains to be elucidated.

The mechanisms differ for glutamate and GABA co-release, presenting an opportunity to dissect their functional roles. Glutamate co-release in the mesoaccumbal pathway occurs in restricted axonal microdomains and is not necessarily packaged within the same synaptic vesicles as dopamine^7^. Conversely, GABA co-release requires the vesicular monoamine transporter-2 (VMAT2), which is also required for dopamine vesicular loading, suggesting that GABA and dopamine can be packaged within the same synaptic vesicles at mesoaccumabal terminals^5^. Dopaminergic neurons do not express conventional GABA synthesizing enzymes, but instead actively uptake ambient GABA in their terminals within the NAc, a process mediated by GABA transporter-1 (GAT1)^5^,^8^.

Here, we describe our efforts to optogenetically dissect the underlying causes of disrupted reward-seeking behaviours in mice that lack the maternal copy of *Ube3a*, a mouse model for Angelman syndrome (*Ube3a*^*m−/p+*^). In doing so, we serendipitously discovered that the loss of the E3-ubiquitin ligase, *Ube3a*, did not directly alter VTA-to-NAc dopamine release but is essential for GABA co-release and the modulation of positively reinforced behaviour. Our findings reveal a novel molecular mechanism underlying GABA co-release from mesoaccumbal terminals and provide new insights into the relevance of this non-canonical mode of neurotransmission to motivated behaviour.

## Results

### Mesoaccumbal optical stimulation in a *Ube3a*-null model

**Figure 1:**
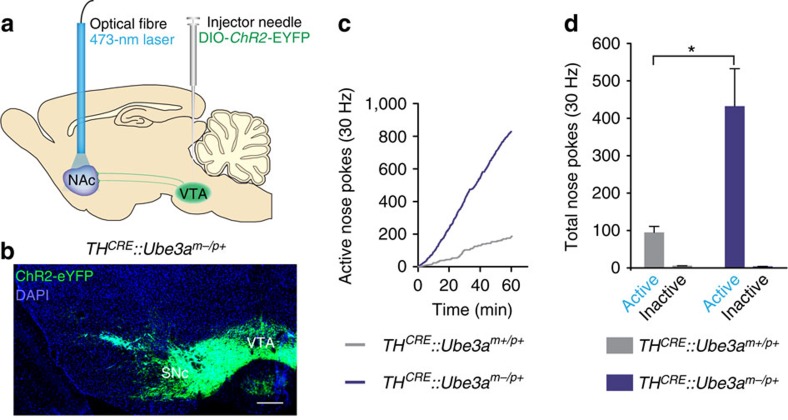
*Ube3a*^*m−/p+*^ mice are hyper-motivated to self-stimulate TH-positive VTA-to-NAc terminals. (**a**) Schematic representation of DIO-ChR2-eYFP viral transduction within the VTA along with NAc chronic fibre placements into *TH*^*CRE*^-positive mice. (**b**) Immunohistochemistry of ChR2-eYFP (green), and DAPI (blue) in a *TH*^*CRE*^*::Ube3a*^*m−/p+*^ mouse. Scale bar, 500 μm. (**c**) Cumulative response plots showing nose pokes that trigger a 30 Hz, 473 nm stimulus (active nose pokes) in representative mice. (**d**) Average number of nose pokes for triggering (active nose pokes) or not triggering (inactive) 30 Hz optical stimulation across a 60-min session. *TH*^*CRE*^*::Ube3a*^*m−/p+*^ mice were significantly more motivated to trigger optical stimulation than *TH*^*CRE*^*::Ube3a*^*m+/p+*^ mice (*n*=7/group, one-way ANOVA, *P<0.05). All bars represent the mean±s.e.m.

In an effort originally designed to elucidate mechanisms underlying dopaminergic dysfunction in an Angelman syndrome mouse model^9^, we used a reductionist approach to selectively manipulate the mesoaccumbal dopaminergic pathway. To target catecholaminergic neurons, mice expressing CRE recombinase within TH^+^ neurons (*TH*^*CRE*^) were crossed into *Ube3a*^*m−/p+*^ mice or their wild-type littermates (*Ube3a*^*m+/p+*^). To specifically manipulate the axon terminals of NAc-projecting TH^+^ neurons, we transduced CRE-dependent AAV5-channelrhodopsin-2 (H134R) fused to enhanced yellow fluorescent protein (ChR2-eYFP) into the VTA and implanted an optical fibre above the NAc (Fig. 1a). We observed qualitatively similar viral expression within *Ube3a*-deficient lines compared with their wild-type littermates (Fig. 1b and Supplementary Fig. 1), but observed no expression following viral injection into CRE-negative control mice. To determine if there are differences in positive-reinforcement behaviour, we trained *TH*^*CRE*^*::Ube3a*^*m−/p+*^ and *TH*^*CRE*^*::Ube3a*^*m+/p+*^ mice to nose-poke for optical stimulation with a fixed-ratio one schedule of reinforcement. We used a 30 Hz optical-stimulation paradigm, as this stimulation frequency is within a range previously shown to produce robust DA release at NAc terminals, and has been used across various behavioural paradigms examining the effects of neuromodulatory release *in vivo*^10^,^11^,^12^,^13^,^14^. *TH*^*CRE*^*::Ube3a*^*m−/p+*^ and *TH*^*CRE*^*::Ube3a*^*m+/p+*^ mice similarly learned an appetitive nose-poke operant task (Supplementary Fig. 2) for natural rewards. However, *TH*^*CRE*^*::Ube3a*^*m−/p+*^ mice nose-poked to receive optical stimulation significantly more than *TH*^*CRE*^*::Ube3a*^*m+/p+*^ mice (Fig. 1c,d). These data suggest that the loss of UBE3A enhances motivation driven through TH^+^ terminals within the NAc, thus increasing optical self-stimulation.

### Motivation and dopaminergic physiology in *Ube3a* ^*m−/p+*^ mice

**Figure 2:**
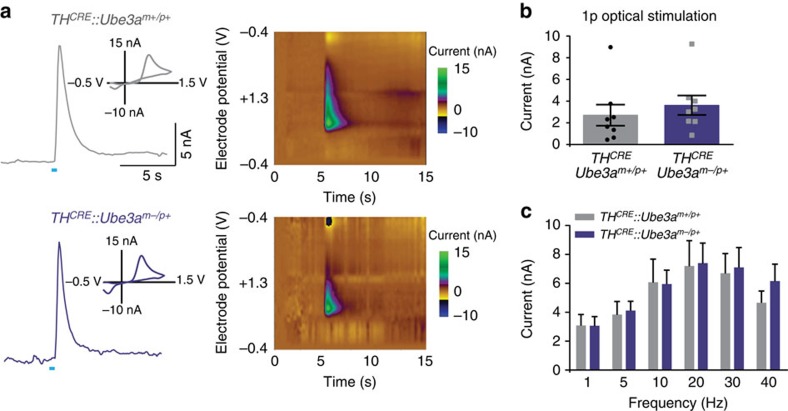
Optically evoked dopamine release is similar in TH-positive VTA-to-NAc terminals in *Ube3a*^*m−/p+*^ and *Ube3a*^*m+/p+*^ mice. (**a**) Representative fast-scan voltammetric recordings from ventral striatal slices in both *TH*^*CRE*^*::Ube3a*^*m−/p+*^ and *TH*^*CRE*^*::Ube3a*^*m+/p+*^ mice. Insets represent background-subtracted electrochemical signal characteristic of oxidized dopamine. Right: consecutive background-subtracted voltammogram recorded over an 8-s interval. Applied electrode potential (E_apps_ versus Ag/AgCl reference electrode) is shown versus time. (**b**) Light-evoked current is similar in both genotypes at 1 pulse (Student's *t*-test, *P*=0.99, *n*=8, 9) and (**c**) across a range of frequencies (one-way ANOVA, *P*=0.83, *n*=11, 12).

**Figure 3:**
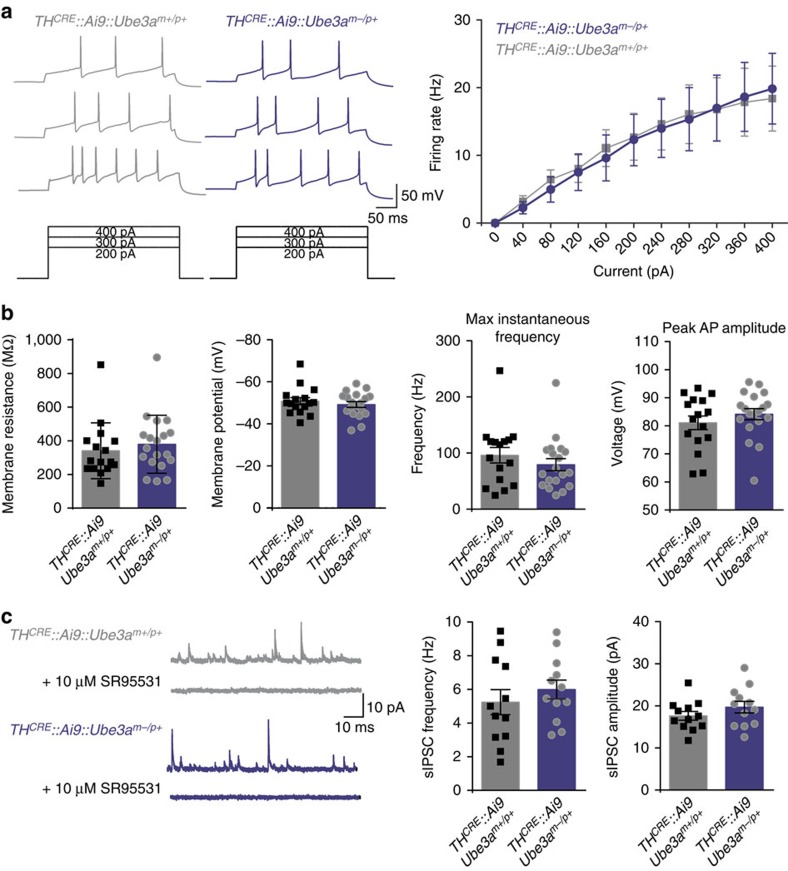
Maternal deletion of *Ube3a* has no apparent effect on intrinsic excitability and inhibitory input onto VTA neurons. (**a**) Representative traces and average data showing action potential firing rates to increasing current injections in Ai9-positive VTA neurons in *TH*^*CRE*^*::Ai9::Ube3a*^*m−/p+*^ and *TH*^*CRE*^*::Ai9::Ube3a*^*m+/p+*^ mice (*n*=18 per group, Student's *t*-test, *P*=0.70). (**b**) Average values of resting membrane potential (Student's *t*-test, *P*=0.46), membrane resistance (*P*=0.25), maximum instantaneous firing frequency (Student's *t*-test, *P*=0.22), and average action potential peak amplitude (Student's *t*-test, *P*=0.32) of Ai9-positive neurons. (**c**) Representative traces and average data showing the frequency and amplitude of sIPSCs in Ai9-positive neurons (*n*=12 for each genotype, Student's *t*-test, *P*=0.42, *P*=0.24). GABAergic currents were validated by bath application of SR95531. All bars represent mean±s.e.m.

On the basis of previous findings^9^, we hypothesized that UBE3A loss might enhance optically evoked reward-seeking by increasing VTA-to-NAc dopamine release. To examine this possibility, we performed *in vitro* fast-scan cyclic voltammetry in brain slices from *TH*^*CRE*^*::Ube3a*^*m−/p+*^ and *TH*^*CRE*^*::Ube3a*^*m+/p+*^ mice expressing ChR2-eYFP within VTA-to-NAc terminals. To probe for possible changes in dopamine release, we optically stimulated either with a single pulse (Fig. 2b) or with stimulation trains across a range of frequencies (Fig. 2c). Contrary to our initial hypothesis, the loss of UBE3A had no effect on dopamine availability or release. Moreover, using *in vitro* whole-cell electrophysiology, we found that intrinsic excitability and inhibition onto VTA *TH*^*CRE+*^ neurons were unchanged in *TH*^*CRE*^*::Ube3a*^*m−/p+*^ mice compared with controls (Fig. 3). Collectively, these findings suggest that enhanced reward seeking in *Ube3a*^*m−/p+*^ mice is not due to changes in dopamine release from VTA-to-NAc dopaminergic terminals or due to altered excitability of dopaminergic VTA neurons in *Ube3a*^*m−/p+*^ mice.

### Consequences of selective *Ube3a* deletion in TH^+^ neurons

**Figure 4:**
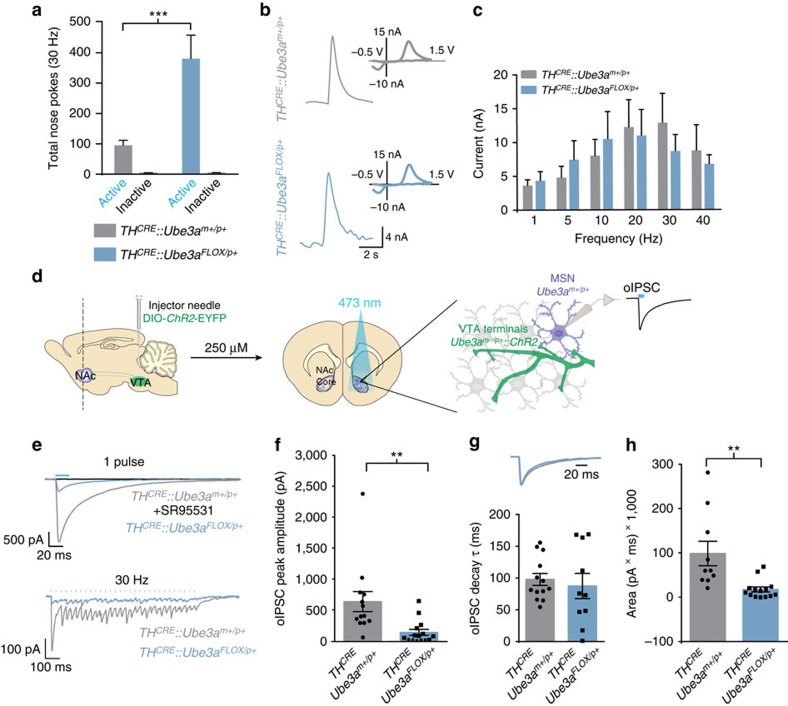
Deleting *Ube3a* in TH-positive neurons decreases GABA co-release and enhances motivational behaviour. (**a**) Average nose pokes for inactive and active ports triggering 30 Hz optical intracranial self-stimulation in a 60 min behavioural session (*n*=5 and 7, one-way ANOVA *P<0.02). Experimental design was similar to that schematized in Fig. 1a, except that *TH*^*CRE*^*::Ube3a*^*FLOX/p+*^ and *TH*^*CRE*^*::Ube3a*^*m+/p+*^ mice were examined to selectively delete *Ube3a* and optically stimulate TH^+^ VTA-to-NAc terminals. (**b**) Representative fast-scan cyclic voltammograms assessing dopamine release within the ventral striatum of *TH*^*CRE*^*::Ube3a*^*FLOX/p+*^ and *TH*^*CRE*^*::Ube3a*^*m+/p+*^ mice. Dopamine release was evoked by 30 Hz (5-pulses) optical stimulation. Insets represent background-subtracted electrochemical signal characteristic of oxidized dopamine. (**c**) Averaged optically evoked dopamine release at a range of frequencies demonstrates that there are no statistical differences between genotypes (*n*=6 and 7, one-way ANOVA *P*>0.05). (**d**) Schematic representing protocol for whole-cell optical IPSC (oIPSC) recordings within the NAc of *TH*^*CRE*^*::Ube3a*^*FLOX/p+*^ mice. IPSCs were pharmacologically isolated (see the Methods section). (**e**) Representative oIPSC traces measured in *TH*^*CRE*^*::Ube3a*^*FLOX/p+*^ and *TH*^*CRE*^*::Ube3a*^*m+/p+*^ mice evoked by single pulses (20 ms) or 30 Hz trains of stimulation. The pharmacological isolation of GABAergic currents was validated by application of the antagonist SR95531. (**f**) Average peak amplitude of single oIPSCs demonstrate a significant reduction of GABAergic currents in *Ube3a*^*FLOX/p+*^ mice (*n*=13 and 15, **P<0.05). (**g**) Average decay kinetics of oIPSCs analysed in **f** (Student's *t*-test, *P*=0.61). Representative traces are shown with normalized amplitude to demonstrate similar current decay kinetics. (**h**) Average oIPSC charge evoked using a similar 30 Hz optical stimulation paradigm used behaviourally (see **a**) and tested *in vitro* (bottom traces of **e**). *TH*^*CRE*^*::Ube3a*^*FLOX/p+*^ mice showed a significant decrease in GABA co-release (*n*=10 and 14, Student's *t*-test **P<0.05). All bars represent the mean±s.e.m.

Because VTA neurons exhibited typical excitability and retain a normal capacity to release dopamine in *Ube3a*^*m−/p+*^ mice (Figs 2 and 3), we questioned if maternal *Ube3a* deletion selectively in catecholaminergic neurons would be sufficient to alter motivational drive. To test this, we used a novel conditional *Ube3a* knockout mouse (*Ube3a*^*FLOX/p+*^) to selectively delete maternal *Ube3a* in a *TH*^*CRE*^-dependent manner (Supplementary Figs 1 and 3a). We injected *TH*^*CRE*^*::Ube3a*^*FLOX/p+*^ and *TH*^*CRE*^*::Ube3a*^*m+/p+*^ mice with CRE-dependent ChR2-eYFP into the VTA to determine if deletion of *Ube3a* within TH^+^ neurons would be sufficient to phenocopy self-stimulation phenotypes observed in AS model mice. Mice were trained to nose-poke (as described above) for optical stimulation of CRE^+^ terminals within the NAc. *TH*^*CRE*^*::Ube3a*^*FLOX/p+*^ mice poked significantly more for 30 Hz stimulation than *TH*^*CRE*^*::Ube3a*^*m+/p+*^ mice (Fig. 4a). This difference occurred in the absence of observable changes in optically evoked dopamine release (Fig. 4b,c). These data demonstrate that the selective loss of UBE3A in TH^+^ neurons is sufficient to enhance motivational drive despite the lack of a detectable deficit in NAc dopamine release.

Dopaminergic terminals in the NAc are also capable of releasing glutamate and GABA^5^,^6^,^7^,^15^. Thus, we tested whether *Ube3a* loss in TH^+^ neurons could alter transmitter co-release. To assess this, we first optogenetically activated VTA-to-NAc terminals and measured GABAergic currents in ventral striatal medium spiny neurons while blocking glutamatergic responses with AMPA and NMDA receptor antagonists (Fig. 4d). We applied a single light-pulse (20 ms) to measure peak amplitude and the kinetics of the resulting current by averaging ≥6 consecutive traces (Fig. 4e). We confirmed *post hoc* that these currents were GABAR-mediated by bath applying SR95531 (10 μM), a selective GABA-A receptor antagonist (Fig. 4e). To verify that these GABAergic currents were a consequence of co-release rather than release by GAD1^+^/TH^−^ neurons spuriously expressing ChR2 due to non-specific CRE expression in the *TH*^*CRE*^ line, we treated mice with the VMAT2 inhibitor, reserpine, thereby selectively inhibiting vesicular loading in dopaminergic terminals capable of co-release. Similar to previous electrophysiological studies^15^ and our own anatomical evidence showing that the vast majority of ChR2-expressing neurons in the VTA are TH^+^ (Supplementary Fig. 4a,b), we found that reserpine abolished nearly all optically evoked GABAergic currents in the NAc (Supplementary Fig. 4c). This further demonstrates that our optical stimulation almost exclusively evoked GABA release from dopaminergic terminals.

Despite exhibiting normal glutamatergic co-release with optical stimulation of VTA-to-NAc terminals (Supplementary Fig. 5a,b), *TH*^*CRE*^*::Ube3a*^*FLOX/p+*^ mice showed a >50% reduction in peak amplitude of GABAergic currents relative to controls, while current decay kinetics proved normal (Fig. 4e–g). Using an optical-stimulation paradigm similar to that used in the behavioural experiments, we also found that *TH*^*CRE*^*::Ube3a*^*FLOX/p+*^ mice exhibit diminished GABAergic currents at 30 Hz stimulation of VTA-to-NAc terminals compared with control mice (Fig. 4h).

### Exogenous VGAT reverses UBE3A-deficient phenotypes

**Figure 5:**
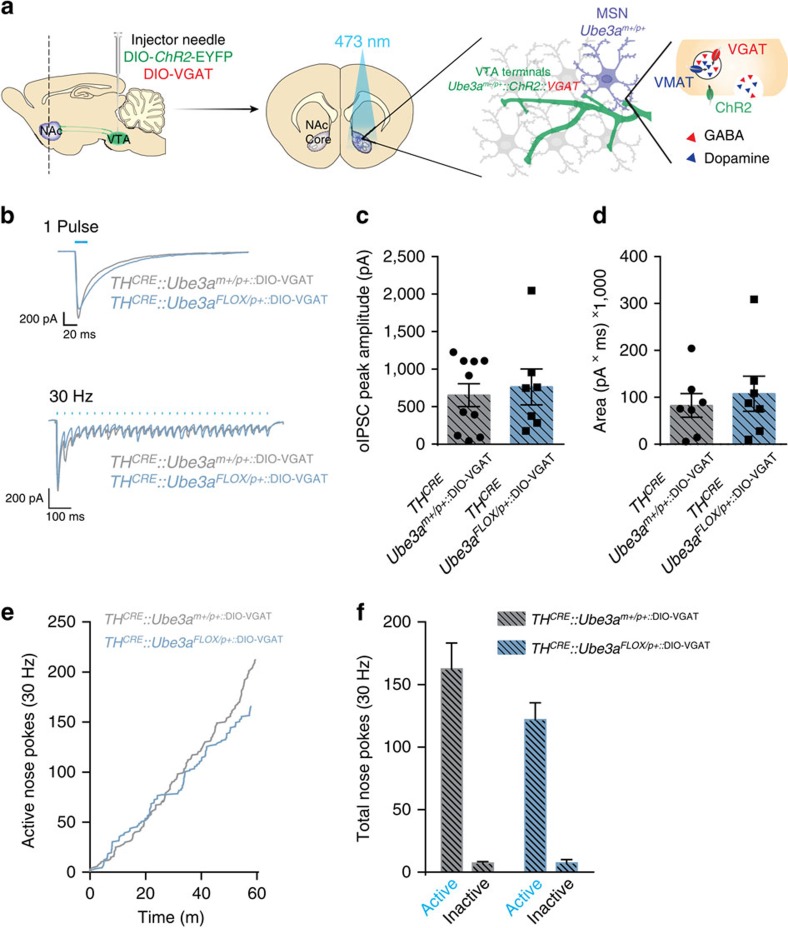
Exogenous VGAT expression in *TH*^*CRE*^**-positive neurons normalizes motivation and GABA co-release in *TH*^*CRE*^*::Ube3a*^*FLOX/p+*^ mice. (**a**) Schematic representation of the experimental paradigm. DIO-VGAT and DIO-ChR2-eYFP were injected in experimental mice to selectively delete *Ube3a* in TH-expressing neurons and to express exogenous ChR2 and VGAT in TH^+^ VTA-to-NAc terminals. (**b**) Representative oIPSC evoked by single pulses (20 ms) or 30 Hz trains of stimulation. (**c**) Average oIPSC peak amplitudes evoked by single pulses (*n*=10 and 7, Student's *t*-test *P*=0.69), and (**d**) average total charge evoked by 30 Hz stimulation (*n*=7 and 7, Student's *t*-test *P*=0.59) demonstrate that exogenous VGAT expression in *TH*^*CRE*^ neurons normalizes GABA co-release between genotypes. (**e**) Response plots showing cumulative nose pokes that trigger a 30 Hz, 473 nm stimulus (active nose pokes) in representative mice. (**f**) Average nose pokes for inactive and active ports triggering 30 Hz optical intracranial self-stimulation in a 60 min behavioural session (*n*=6 and 6, one-way ANOVA *P*=0.12). Bars represent mean±s.e.m.

We tested if GABA co-release could be restored in *TH*^*CRE*^*::Ube3a*^*FLOX/p+*^ mice, and whether this could normalize motivational drive. The observed decrease in GABA co-release could arise from changes in GABA uptake, availability, release or vesicular loading^5^,^15^,^16^. To enhance vesicular loading of GABA in TH^+^ terminals, we used a CRE-dependent virus expressing the vesicular GABA transporter (VGAT) and introduced this with DIO-ChR2-eYFP at a 1:1 ratio within the VTA (Fig. 5a)^15^. GABA co-release evoked by a single light-pulse or a 30 Hz optical stimulation was similar in *TH*^*CRE*^*::Ube3a*^*FLOX/p+::*DIO-VGAT^ mice compared with *TH*^*CRE*^*::Ube3a*^*m+/p+::*DIO-VGAT^ mice (Fig. 5b–d, *P*>0.05). Exogenous expression of VGAT within TH^+^ terminals was also sufficient to normalize optical intracranial self-stimulation responding in *TH*^*CRE*^*::Ube3a*^*FLOX/p+::*DIO-VGAT^ mice compared with *TH*^*CRE*^*::Ube3a*^*m+/p+::*DIO-VGAT^ mice (Fig. 5e,f), suggesting a causal relationship between the observed deficits in GABA co-release and alterations in motivational drive in the absence of UBE3A (Fig. 5f, *P*>0.05).

## Discussion

In this study we optogenetically dissected mesoaccumbal circuitry to investigate the underlying causes of disrupted reward-seeking behaviours in a *Ube3a*-deficient mouse model. Collectively, our data indicate that UBE3A regulates circuits involved in motivated behaviour and, in particular, GABA co-release from putative dopaminergic mesoaccumbal terminals. Following UBE3A loss, suppressed GABA co-release and enhanced optical self-stimulation occur in the absence of other measured cellular and synaptic deficits in the mesoaccumbal pathway. Remarkably, exogenous expression of VGAT is sufficient to reinstate GABA co-release and normalize motivational drive in the UBE3A-deficient circuit, suggesting a causal link between GABA co-release from VTA-to-NAc terminals and the modulation of motivation.

We previously concluded, based on electrical stimulation of the medial forebrain bundle in *Ube3a*^*m−/p+*^ mice, that the loss of UBE3A enhances dopamine release at the mesoaccumbal pathway, thereby reducing reward threshold^9^. Consequently, we hypothesized here that selective loss of UBE3A in TH^+^ neurons would be sufficient to alter dopamine release within the NAc. Instead, we find that optogenetically evoked release in VTA-to-NAc terminals is normal (Figs 2b,c and 4b,c). Given that medial forebrain bundle stimulation is known to activate diverse circuit pathways^17^, including septal nuclei, there are numerous explanations for our previous observation of enhanced dopamine release in the NAc of *Ube3a*^*m−/p+*^ mice^9^. Regardless, our new results demonstrate that the loss of UBE3A specifically in TH-expressing neurons leads to a reduction in GABA co-release without corresponding reductions in dopamine release, and that this reduction of GABA co-release is sufficient to enhance optogenetically mediated, positively reinforced behaviours. Importantly, GABA co-release may also regulate natural appetitive behaviours such as copulation, social interactions and, as shown recently, binge drinking^8^. However, sucrose seeking is unaffected in both *Ube3a*^*m−/p+*^ and *TH*^*CRE*^*::Ube3a*^*FLOX/p+*^ (Supplementary Figs 2 and 5c), indicating that GABA co-release is not involved in food-seeking and suggests at least some degree of specificity for this release mechanism in the modulation of appetitive behaviour.

While these data provide the first functional role for GABA co-release within the ventral striatum in an *in vivo* context, the utility for GABA co-release regulating motivational drive for ethologically relevant appetitive stimuli remains to be determined. Furthermore, we cannot rule out the possibility that our effect might be driven in part by ectopic CRE expression within GABAergic neurons^18^. However, this possibility seems unlikely given that we observe a low percentage of ChR2 and GAD1 overlap (∼3%), and that we also found that the VMAT2 inhibitor (reserpine) blocked most of the GABAergic currents. Moreover, GABAergic VTA-to-NAc projections preferentially synapse on cholinergic interneurons within the NAc^19^, further decreasing the possibility of ectopic CRE expression contributing to our observed effects.

UBE3A is an E3-ubiquitin ligase that transfers ubiquitin to a targeted substrate for proteasomal degradation^20^, thus UBE3A loss causes an accumulation of targeted UBE3A substrates. Recent evidence suggests that GAT1 is a potential UBE3A substrate^16^, proposing that UBE3A loss could directly alter GAT1 transport function. While the mechanistic details remain to be established, our findings support the hypothesis that anomalous GABA uptake on UBE3A loss not only leads to severely attenuated GABA co-release, but also augments motivational drive via mesoaccumbal pathway activation.

## Methods

### Experimental subjects and stereotaxic surgeries

*Cg-Tg-TH:Cre* mice (JAX #: 008601), *Rosa26-stop-floxed-tdTomato* (Ai9, JAX #: 007909) and *Ube3a*-deficient (JAX #: 016590) mice were obtained through Jackson Laboratories (Bar Harbor, ME). *Ube3a-floxed* mice (*Ube3a*^*FLOX*^) were engineered in conjunction with the UNC Animal Models Core. Briefly, C57BL/6 mouse embryonic stem cells were electroporated with an AsiSI-linearized *Ube3a*^*KO1st*^ targeting construct, which was generated by the trans-NIH Knockout Mouse Project (KOMP) and obtained from the KOMP repository (www.komp.org). To produce *Ube3a*^*KO1st*^-targeted chimeric mice, *Ube3a*^*KO1st*^-targeted embryonic stem cells were microinjected into C57BL/6-albino blastocysts. Resultant germline chimeric males (determined by the transmission of coat colour in parallel breeding) were then crossed to C57BL/6 female homozygous *Rosa26-FLPe* mice (009086, Jackson Laboratories) in order to excise the *FRT*-flanked lacZ gene trap from the *Ube3a*^*KO1st*^ allele and thereby produce the *Ube3a*^*FLOX*^ allele. *Ube3a*^*FLOX*^ mice were genotyped using the following PCR primers: *Ube3a*^*FLOX*^ F (5′-AAAATTGGGTATGCGAGCTG-3′) and *Ube3a*^*FLOX*^ R (5′-GGGGTCTAAGGGCCTATGAA-3′).

All mice were maintained on a congenic C57BL/6 background, had *ad libitum* access to food and water, and were housed on a 12:12 light:dark cycle. Mice for electrophysiological recordings were aged P60–P90 and were compared with wild-type age- and sex-matched controls. The experimenter was blind to genotype, and littermate controls were used when possible. Behavioural mice were group housed until surgery, which was performed when mice weighed 25–30 g (∼P60). Mice were anesthetized with ketamine (150 mg kg^−1^) and xylazine (50 mg kg^−1^), and then placed in a stereotaxic frame (Kopf Instruments) for bilateral injections (0.5 μl) of purified adeno-associated virus (∼10^12^ viral genomes ml^−1^, packaged and titered by the UNC Viral Vector Core Facility) into the VTA (coordinates from bregma: −3.15 anterior/posterior, ±0.75 medial/lateral, −4.75 dorsal/ventral). VTA neurons in *TH*^*CRE*^-positive *Ube3a*^*m−/p+*^, *Ube3a*^*FLOX/p+*^ or *Ube3a*^*+/+*^ mice were transduced with virus encoding ChR2-eYFP and/or VGAT under the control of the *EF1α* promoter. DIO-VGAT was generously provided by the laboratory of Bernardo Sabatini^15^. Mice were individually housed following surgery. For behavioural experiments, mice were implanted with bilateral chronic optical fibres directed above the NAc (coordinates from bregma: +1.2 A/P, ±1.6 M/L, −4.6 D/V at a 10° angle). We performed all experiments 5–8 weeks post-surgery. All procedures were conducted in accordance with the Guide for the Care and Use of Laboratory Animals as adopted by the National Institutes of Health, and with approval of the UNC Institutional Animal Care and Use committees.

### Slice preparation for whole-cell electrophysiology and voltammetry

Mice were anesthetized with pentobarbital (40 mg kg^−1^) and intracardially perfused with ice-cold dissection buffer (in mM: 87 NaCl, 2.5 KCl, 1.25 NaH_2_PO_4_, 26 NAHCO_3_, 75 sucrose, 10 dextrose, 1.3 ascorbic acid, 7 MgCl_2_ and 0.5 CaCl_2_) bubbled with 95% O_2_–5% CO_2_ after disappearance of corneal reflexes. Brains were then rapidly removed and immersed in ice-cold dissection buffer. VTA sections were dissected and 200-μm thick horizontal slices were prepared using a vibrating microtome (Leica VT1200S). NAc sections were dissected and 250-μm thick coronal slices were prepared as described within the VTA. Slices recovered for 20 min in a 35 °C submersion chamber filled with oxygenated artificial cerebrospinal fluid (ACSF; in mM: 124 NaCl, 3 KCl, 1.25 NaH_2_PO_4_, 26 NAHCO_3_, 1 MgCl_2_, 2 CaCl_2_ and 20 glucose) and then kept at room temperature for >40 min until use^21^.

### Voltage-clamp recordings

To isolate sIPSCs (spontaneous inhibitory postsynaptic currents), slices were placed in a submersion chamber, maintained at 27 °C and perfused at 2 ml min^−1^ with oxygenated ACSF (as described above) and held at the AMPAR reversal potential (+10 mV). AMPAR reversal potential was empirically determined by applying a series of 10 pA current injections (−70 to +60 mV) in the presence of picrotoxin and D,L-APV. sIPSCs were confirmed *post hoc* by the addition of 10 μM SR95531 (Abcam). Cells were visualized using a Zeiss Examiner microscope equipped with infrared differential interference contrast optics. Putative VTA-to-NAc dopaminergic neurons were identified by *tdTomato* fluorescence medial to the medial terminal nucleus of the accessory optic tract in *TH*^*CRE*^*::Ai9::Ube3a*^*m−/p+*^ or wild-type mice. Patch pipettes were pulled from thick-walled borosilicate glass (P2000, Sutter Instruments Novato, CA). Open-tip resistances were between 2.5–5 MΩ and were backfilled with an internal containing (in mM): 100 CsCH_3_SO_3_, 15 CsCl, 2.5 MgCl_2_, 10 Hepes, 5 QX-314, 5 BAPTA, 4 Mg-ATP, 0.3 Mg-GTP and 0.025 Alexa-488 with pH adjusted to 7.25 with 1 M CsOH and osmolarity adjusted to ∼295 mOsM by the addition of sucrose. Voltage-clamp recordings were performed in the whole-cell configuration using a patch-clamp amplifier (Multiclamp 700B, Molecular Devices), and data were acquired and analysed using pClamp 10 software (Molecular Devices). Pipette seal resistances were >1 GΩ, and pipette capacitive transients were minimized before breakthrough. Changes in series and input resistance were monitored throughout the experiment by giving a test pulse every 30 s and measuring the amplitude of the capacitive current. Cells were discarded if series resistance rose above 30 MΩ.

Widefield ChR2-mediated photostimulation was provided through a × 20/0.8 NA objective using single-photon excitation through a 470 nm λ-filter. Light power was provided by a Lambda DG-4 300 W Xenon bulb (Sutter Instruments). This light was coupled to a Mosaic microelectro-mechanical-system digital micromirror device (Andor Technology) and was shuttered via pClamp-delivered TTL pulse to the Lambda DG-4 as previously described^22^. Co-release from TH-positive terminals originating in the VTA was measured in medium spiny neurons in response to a single 20 ms pulse as well as to a 1 s train of pulses at 30 Hz stimulation. Medium spiny neurons were identified by their shape and passive membrane properties (C_m_, R_m_ and decay constant) measured immediately after break-in in the voltage-clamp configuration holding at −70 mV.

Activation of ChR2-expressing fibres was performed by using square illumination patterns (as described in ref. 22) in animals previously used in the optical intracranial self-stimulation paradigm. In response to these stimuli, mean E/IPSC amplitude, decay/rise tau and total charge were measured by averaging ≥6 consecutive traces. Optical inhibitory/excitatory postsynaptic currents (oIPSCs/oEPSCs) were measured in ventral striatum medium spiny neurons and were selected by their membrane properties. oIPSCs were isolated by including DNQX (20 μM, Abcam) and DL-APV (100 μM, Abcam) in the external solution, whereas oEPSCs were isolated by including picrotoxin (50 μM, Sigma) in the external solution. Patch pipette open-tip resistances were between 2.5–6 MΩ and were backfilled with (in mM): 125 CsCl, 10 TEA-Cl, 0.1 EGTA (CsOH), 10 Hepes, 3.3 QX-314, 1.8 MgCl_2_, 4 ATP, 0.3 GTP, 8 Na_2_-Phosphocreatine with pH adjusted to 7.25 with 1 M CsOH and osmolarity adjusted to ∼295 by the addition of sucrose for oIPSC experiments or with (in mM): 100 K-gluconate, 20 KCl, 10 Hepes, 0.2 EGTA, 4 ATP, 0.3 GTP, 10 Na_2_-Phosphocreatine with pH adjusted to 7.25 with 1 M KOH and osmolarity adjusted to ∼295 by the addition of sucrose for oEPSC experiments. The high internal-chloride concentration increased the chloride-driving force and allowed for oIPSCs to be more easily resolved at −70 mV. Changes in series and input resistance were monitored throughout the experiment and did not differ between genotypes (*P*>0.05). Recordings were discarded if series resistance rose above 30 MΩ.

For validation of VMAT2-mediated GABA co-release from TH^+^ neurons, mice were injected intraperitoneally 24 h before killing with reserpine (5 mg kg^−1^, Tocris 2742). Mice were then killed if they displayed ptosis and paralysis the following day. Slices of ventral striatum were prepared as described above. Reserpine was also included within the slice recovery chamber (1 μM), and slices were constantly incubated in reserpine before recording. Recordings of GABA co-release were performed as described above.

### Current-clamp recordings

Intrinsic excitability experiments were performed at −50 to −60 mV in ACSF containing picrotoxin (50 μM, Sigma), DNQX (20 μM, Abcam) and DL-APV (100 μM, Abcam) to block excitatory and inhibitory transmission. Putative VTA-to-NAc dopaminergic cells were selected as described above and pipettes were backfilled with (in mM): 100 K-gluconate, 20 KCl, 10 Hepes, 0.2 EGTA, 4 ATP, 0.3 GTP, 10 Na_2_-Phosphocreatine and 0.015 Alexa-488 (Life Technologies) with pH adjusted to 7.25 with 1 M KOH and osmolarity adjusted to ∼295 by the addition of sucrose. For frequency–current plots, current was injected at 40 pA steps and average action potential frequency was calculated. Peak amplitude was calculated by averaging the max amplitude for all events across all collected traces. Maximum instantaneous frequency was calculated by taking the inverse of the shortest inter-event interval across all collected traces. Changes in series and input resistance were monitored throughout the experiment by giving a test pulse every 30 s and measuring the amplitude of the capacitive current. Cells were discarded if series resistance rose above 30 MΩ.

### Fast-scan cyclic voltammetry

Ventral striatum sections were prepared as described above. Electrochemical data were acquired using a custom-written software in LabVIEW (Tar Heel CV) and filtered offline at 1 kHz. Briefly, carbon-fibre microelectrodes (50 μM in length) were scanned from −0.4 V to 1.3 V at a rate of 400 V s^−1^. Samples were acquired at a rate of 10 Hz. Light pulses (5 ms, 473 nm, 1 mW) were delivered through a × 40 objective via a high-powered LED (Thorlabs) to evoke dopamine release. A single pulse or 5 light pulses were delivered at 1, 5, 10, 20, 30 or 40 Hz in a randomized order. Immediately after optical stimulation of the slice, background-subtracted cyclic voltammograms were generated, which were characteristic of dopamine (peak oxidation potential of 600–700 mV).

### Immunohistochemistry

Mice were anesthetized with pentobarbital and then perfused with 4% paraformaldehyde in phosphate-buffered saline (PBS; pH ∼7.3). Samples were placed in 10, 20 and then 30% sucrose in PBS before being cut at 40 μm using a cryostat (Leica). Sections were collected, rinsed (PBS) and blocked with 5% normal goat serum and 0.2% Triton X-100 in PBS. Sections were then tumbled in this blocking solution with primary antibody for 24 h at 4 °C. The primary antibodies used in this study were rabbit anti-TH (1:650 Millipore, AB152), chicken anti-EGFP (1:1,000 Aves) and mouse anti-UBE3A (1:750 Sigma clone 3E5, SAB1404508). Transgenic fluorescent proteins expressed via *CRE*-mediated recombination (Ai9 mice) were not further antibody enhanced. Secondary detection was performed with Alexa Fluor 488, 568 or 633 conjugated goat anti-rabbit, anti-chicken or anti-mouse antibodies (Invitrogen). Mounted sections were imaged on a Zeiss LSM 710 Confocal Microscope using × 20/0.8 or × 40/1.3 NA objectives. Immunohistochemistry was used to validate viral injection, fibre placement and recombination efficiency *post hoc* in experimental animals.

### Fluorescence *in situ* hybridization

Mice were rapidly decapitated after 2 weeks post-operationally and brains were snap frozen in dry ice in O.C.T. Compound (Fisher Scientific). Fresh, frozen brains were sectioned at 20 μm on a cryostat (CM1950, Leica) onto charged slides (Leica). Samples were hybridized to *GAD1* antisense or sense riboprobes. A 950-bp riboprobe complementary to *GAD1*-sense cDNA was inserted into the pcrII-TOPO vector (Life Technologies). Plasmid DNA was then digested with *Eco*RV or *Asp*718 in order to create sense and antisense template for *in vitro* transcription. All probes were created using florescein-labelled nucleotides for detection. *Eco*RV template was transcribed with Sp6 RNA polymerase for the generation of the sense riboprobe and *Asp*718 template was transcribed with T7 RNA polymerase for the generation of the antisense riboprobe. Fluorescence *in situ* hybridization was performed at room temperature unless otherwise specified. Tissue was dried at 50 °C, fixed in 4% DEPC-PFA for 15 min and washed in DEPC-PBS three times for 5 min. The tissue was then acetylated in 1 × triethanolamine-HCl with 0.25% acetic anhydride for 10 min and subsequently washed in DEPC-PBS 3 times for 5 min each. Next, the tissue was pre-hybridized for 3 h at 65 °C in hybridization buffer containing 5 × saline sodium citrate (SSC), 50% formamide, 1 mg ml^−1^ yeast tRNA, 0.1 mg ml^−1^ heparin, 0.1% tween-20, 0.005 M EDTA (pH 8.0) and 0.1% CHAPS. Subsequently, the tissue was incubated in hybridization buffer containing a probe for *GAD1* (fluorescein-labelled). Post-hybridization stringency washes were performed sequentially at 65 °C in pre-warmed buffers: 1 × 15 min in 2 × SSC and 3 × 20 min in 0.2 × SSC buffer. After the stringency washes, tissue was washed additionally at room temperature 2 × 10 min in TS7.5 (0.1 M Tris-HCl, pH 7.5, 0.15 M NaCl). Tissue was then incubated in 3% H_2_O_2_ in methanol and washed 3 × 5 min in TS7.5 to quench endogenous hydrogen peroxidase activity. Sections were then incubated for 1 h in 1% blocking buffer (Perkin Elmer), followed by incubation for 24 h at 4 °C in anti-Fluorescein-POD (1:350). Then, sections were washed 3 × 10 min in TNT wash buffer (0.1 M Tris-HCl (pH 7.5), 0.15 M NaCl, 0.05% Tween-20), sections underwent a tyramide signal amplification with TSA plus TM POD-DNP (Perkin Elmer, NEL747B) 1:50 in amplification diluent. Following a 4–7 min incubation, sections were washed with TNT wash buffer 4 × 10 min and incubated in a DNP primary antibody conjugated to Alexa Fluor 488 (1:500, Molecular Probes) at 3 h at room temperature. Sections were then washed 3 × 10 min in PBS and coverslipped with a mounting media containing DAPI (Life Technologies).

### *In vivo* optogenetic stimulation

For all behavioural experiments, mice were injected with AAV5-EF1α-DIO-ChR2-eYFP and/or AAV8-EF1α-DIO-VGAT (at a 1:1 ratio) virus and implanted with bilateral custom-made optical fibre targeted to the NAc core^23^. Mice were connected to a ‘dummy' optical patch cable 5 days before the experiment each day for 60 min to habituate them to the tether procedure. Following the tethering procedure, we ran the mice in several behavioural procedures (detailed below). We used a 10 mW, 473 nm laser with a stimulation frequency of 30 Hz and a 5 ms pulse width duration for all behavioural assays unless otherwise noted.

### Positive-reinforcement procedures and optical self-stimulation

Behavioural training and testing occurred in mouse operant chambers (Med Associates) interfaced with optogenetic stimulation equipment. Behavioural paradigms were performed during their respective dark cycle. Food-restricted male mice (90% of their free-feeding bodyweight) were trained on a fixed-ratio (1:1) training schedule for one session per day for 60 min, in which each nose-poke resulted in 20 μl of a 15% sucrose administration until the number of nose pokes did not vary >20% across 3 consecutive days. In addition, active nose-poke ports were coupled with a cue light that remained on. With each successful nose-poke, the cue light turned off and a tone would be presented for 3 s. Once the mice reached a stable number of nose pokes, they were habituated for 5 consecutive days to the patch cable with optical stimulation (3 s of 30 Hz) time-locked to the cue following each active nose-poke. After the 5-day habituation phase, mice were then tested following a 2-day break. Active and inactive nose pokes were recorded in addition to time-stamps.

### Statistics and data analysis

We plotted all data and performed all statistical analyses using GraphPad Prism software. All graphs are represented as the mean±s.e.m. For statistical analyses, we used two-way analysis of variance (Figs 1d, 2c and 4a), one-way analysis of variance (Figs 2c and 3c) or two-tailed Student's *t*-test (Figs 2b, 3, 4f–h and 5c,d, Supplementary Figs 2b, 3a, 4b,c and 5b,c). Statistical significance is represented as follows: *P<0.05, **P<0.02 and ***P<0.001. Minimum sample sizes were estimated from previously published data sets with similar experimental parameters. The only data points that were discarded were done so before unblinding and only because the data points did not meet *a priori* criteria for data inclusion (for example, series resistance in a whole-cell recording was above our established limit for inclusion). No outlier test was used to discount any data point, and all data points are included within the summarized graphs.

## Additional information

**How to cite this article:** Berrios, J. *et al*. Loss of UBE3A from TH-expressing neurons suppresses GABA co-release and enhances VTA-NAc optical self-stimulation. *Nat. Commun.* 7:10702 doi: 10.1038/ncomms10702 (2016).

## Supplementary Material

Supplementary Information Supplementary Figures 1-5
