# Supplementary material for: Loss of UBE3A from TH-expressing neurons suppresses GABA co-release and enhances VTA-NAc optical self-stimulation
Source: Nat Commun. 2016 Feb 12;7:10702. doi: 10.1038/ncomms10702 (PMC4754338; doi:10.1038/ncomms10702)
Supplement: Supplementary Information — Supplementary Figures 1-5 [file ncomms10702-s1.pdf]

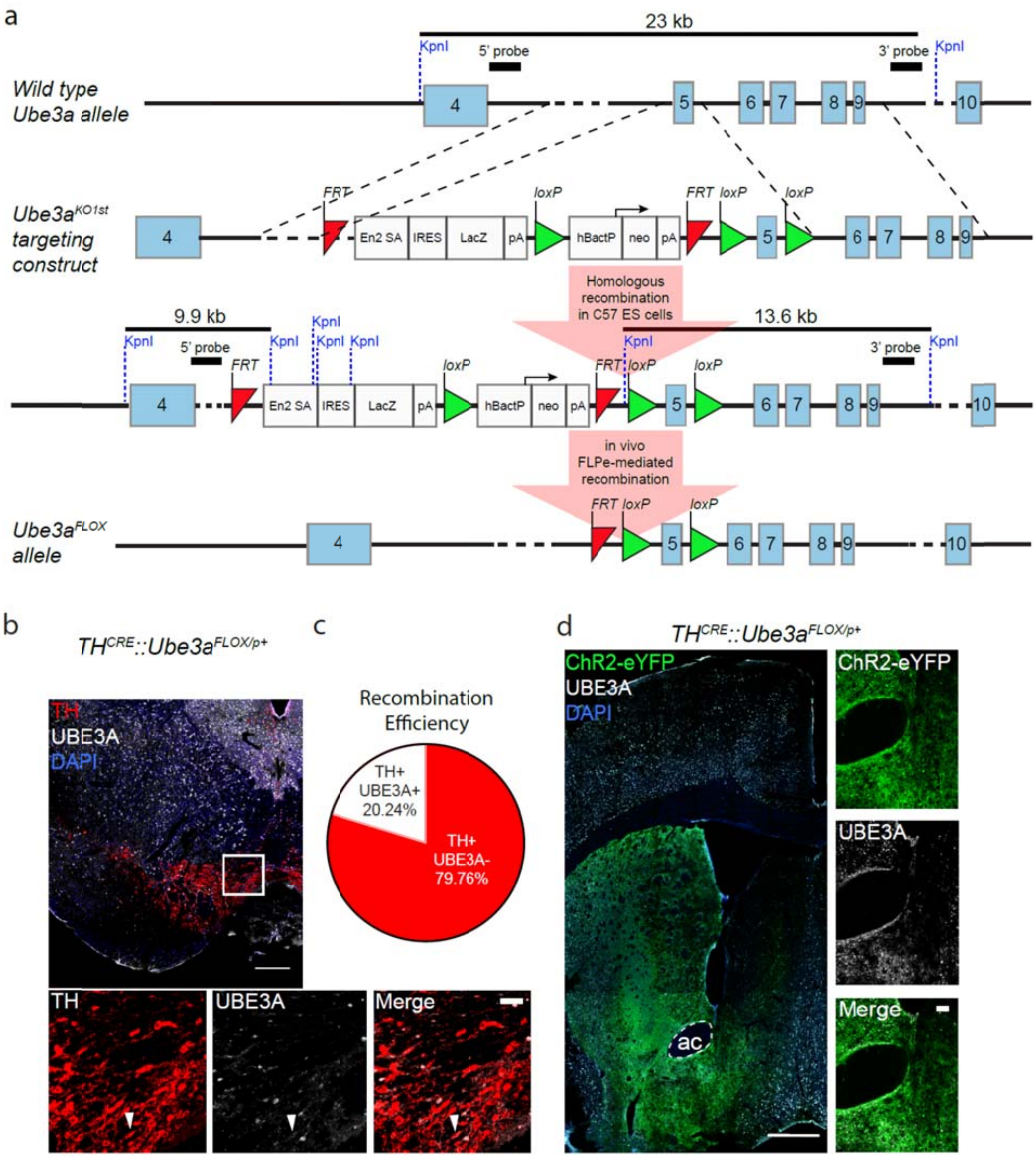

Supplementary Figure 1: *TH*^{CRE}-mediated recombination of the maternally inherited *Ube3a*^{FLOX} allele abolishes UBE3A within VTA but not striatal neurons

(**a**) Schematic of strategy used to generate C57BL/6 mice carrying the *Ube3a*^{FLOX} allele. (**b**) Immunohistochemistry showing UBE3A-positive (white) and TH-positive (red) neurons in *TH*^{CRE}*::Ube3a*$^{FLOX/p+}$ mice. High magnification views are of area outlined by white box. Scale bar = 500 μM (top) or 50 μM (bottom). Arrowhead represents residual UBE3A expression within a TH-positive neuron. (**c**) Quantification demonstrating a recombination efficiency of ~80% (n=2 mice). (**d**) Immunohistochemistry showing ChR2-eYFP (green), UBE3A (white), and DAPI (blue) in the striatum of *TH*^{CRE}*::Ube3a*$^{FLOX/p+}$ mice following injection of DIO-ChR2-eYFP into the VTA. UBE3A expression is qualitatively similar to wild-type levels of UBE3A within the striatum. ac=anterior commissure. Scale bar = 500 μM (left) or 50 μM (right, merge).

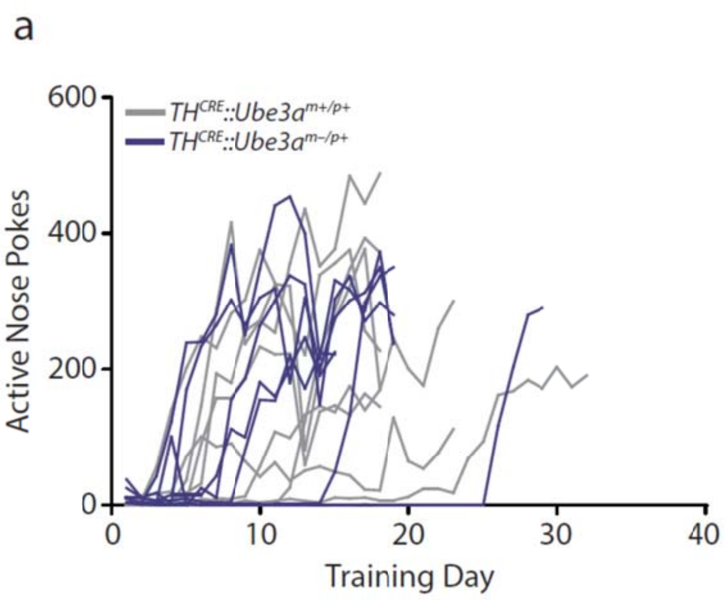

Supplementary Figure 2: *TH^{CRE}::Ube3a$^{m–/p+}$* mice learn on similar timescales

(**a**) Plot showing active nose pokes for each mouse across sucrose training days. n=8 and 7. (**b**) Average number of nose pokes performed on the last sucrose training day for *TH^{CRE}::Ube3a$^{m–/p+}$* and *TH^{CRE}::Ube3a$^{m+/p+}$* mice (student's t-test, p=0.84). Bars represent mean ± SEM.

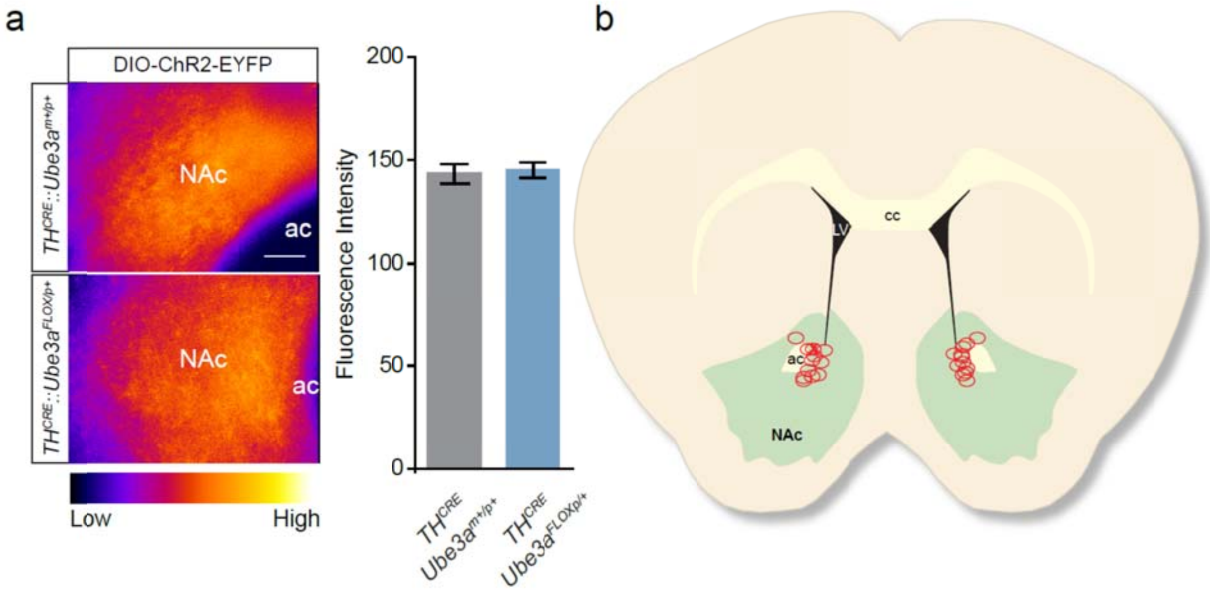

Supplementary Figure 3: ChR2-eYFP terminal expression and optical fiber placements are similar across genotypes

(**a**) Representative images of ventral striatal expression of DIO-ChR2-eYFP in both TH^{CRE}*::Ube3a*$^{m+/p+}$ and TH^{CRE}*::Ube3a*$^{FLOX/p+}$ mice. Fluorescence intensity is represented by a heat plot. Scale bar = 50 μM. Bar graph represents quantification of ventral striatal ChR2-eYFP intensity taken medial to the anterior commissure (student's t-test, p=0.769). (**b**) Optical fiber placements represented by open circles in 12 mice of three different genotypes (TH^{CRE}*::Ube3a*$^{m+/p+}$, TH^{CRE}*::Ube3a*$^{m–/p+}$, and TH^{CRE}*::Ube3a*$^{FLOX/p+}$). There is not a qualitative difference in placements across genotypes. Bars represent mean ± SEM.

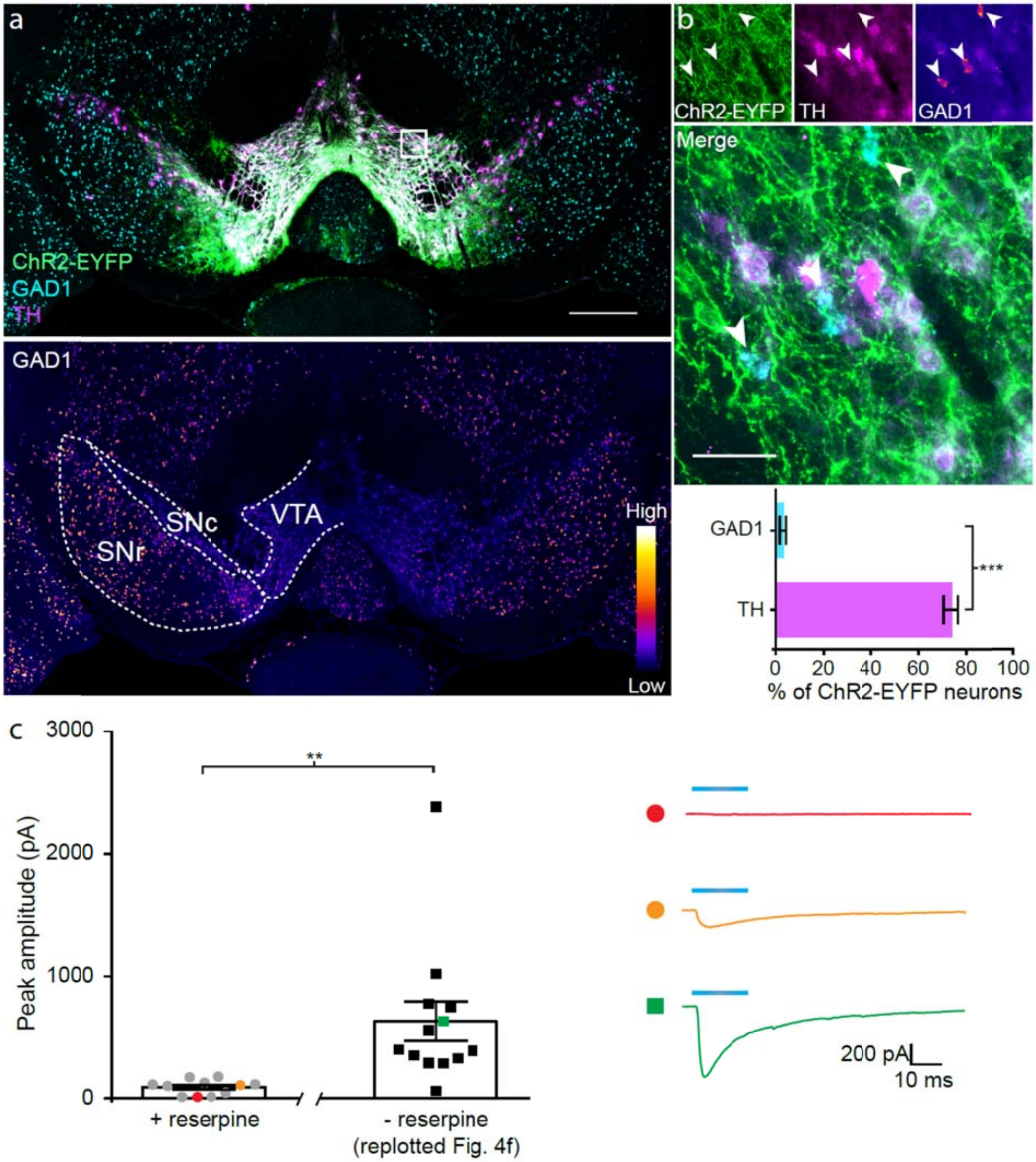

Supplementary Figure 4: *TH*^{CRE} mediated recombination occurs predominantly in TH-positive neurons, and optogenetic stimulation of *TH*^{CRE}-ChR2 positive VTA-to-NAc terminals evokes reserpine-sensitive GABA co-release

(**a**) Low magnification overview of ChR2-eYFP protein (green), TH protein (magenta), and GAD1 mRNA (cyan). Below depicts a heat map of GAD1 expression. Scale bar = 500 µM. (**b**) Max projection of the lateral VTA representing ChR2-eYFP, TH, and GAD1 expression and a larger merged fluorescence image (below). Scale bar = 50 µM. Bar graph shows quantification of ChR2-eYFP co-expression with GAD1 and TH. Three independent animals were sampled. GAD1 mRNA co-expresses with only ~3% of ChR2-eYFP positive somas. (student's t-test, ***p<0.0001). (**c**) Reserpine blocked GABAergic currents in slices from TH^{CRE} mice when compared to untreated slices (n=11 and 13, student's t-test **p<0.05; data from untreated slices are replotted from Fig. 4f), suggesting that the vesicular monoamine transporter VMAT2 is required for GABA co-release in this pathway. Right: Representative traces of responses from neurons in the presence (circles) or absence (squares) of reserpine. All bars represent mean ± SEM.

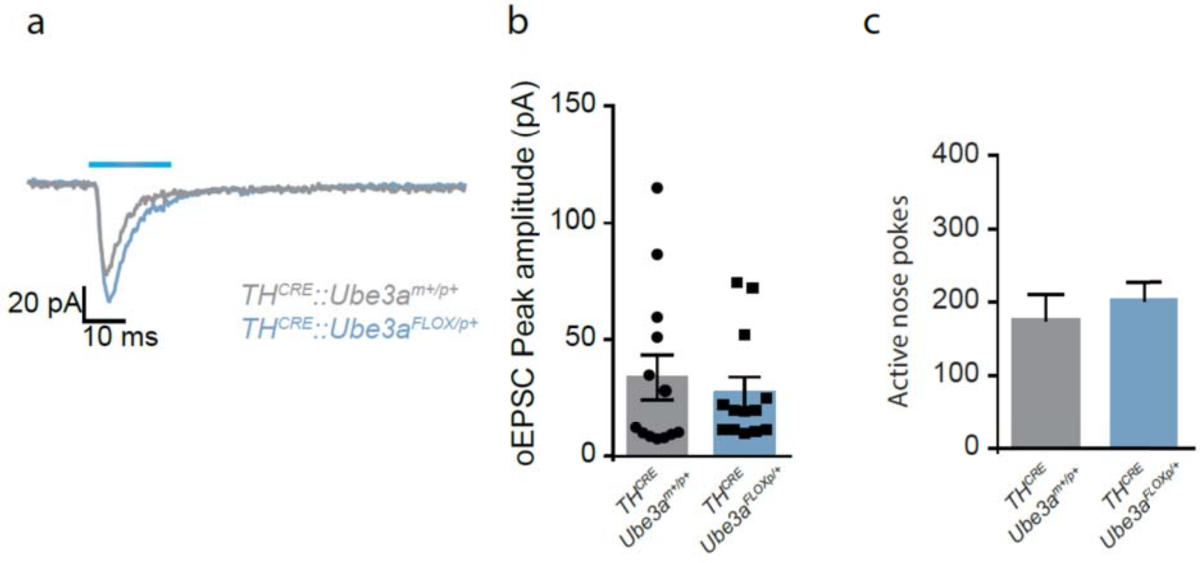

Supplementary Figure 5: Glutamate co-release and sucrose seeking is similar between *TH^{CRE}::Ube3a$^{m+/p+}$* and *TH^{CRE}::Ube3a$^{FLOX/p+}$* mice

(**a**) Representative averaged traces (≥ 6 sweeps) of glutamate co-release from TH-expressing VTA-to-NAc terminals. (**b**) Average peak amplitude of single oEPSCs demonstrate similar excitatory currents in *TH^{CRE}::Ube3a$^{m+/p+}$* and *TH^{CRE}::Ube3a$^{FLOX/p+}$* mice (n=13/genotype, student's t-test, p=0.59). (**c**) Averaged number of active nose pokes for a 1:1 positive reinforcement schedule indicates that sucrose seeking is similar in *TH^{CRE}::Ube3a$^{m+/p+}$* and *TH^{CRE}::Ube3a$^{FLOX/p+}$* mice (n= 9 and 11, student's t-test, p=0.51). All bars represent mean ± SEM.
